# Optimizing cultivation density for *Pseudostellaria heterophylla*: a novel evaluation strategy integrating electrophysiological and traditional traits for early growth indication

**DOI:** 10.3389/fpls.2026.1943951

**Published:** 2026-10-09

**Authors:** Xiaolong Li, Shiyi Pan, Zelan Wang, Chenglin Tang, Bing Tian, Qiuping Wang, Cheng Zhang, Jun Li

**Affiliations:** 1 School of Public Health, State Key Laboratory of Discovery and Utilization of Functional Components in Traditional Chinese Medicine, Key Laboratory of Environmental Pollution Monitoring and Disease of Ministry of Education, Guizhou Medical University, Guiyang, China; 2 Guizhou Crop Technology Extension Station, Guizhou Agricultural Ecology and Resource Protection Station, Agriculture and Rural Affairs Department of Guizhou Province, Guiyang, China; 3 Department of Food and Medicine, Guizhou Vocational College of Agriculture, Qingzhen, China

**Keywords:** comprehensive evaluation, early growth indication, electrophysiological characteristics, planting density, *Pseudostellaria heterophylla*

## Abstract

**Background:**

*Pseudostellaria heterophylla* is an herb with medicinal, edible and ornamental uses, and unreasonable planting density severely restricts its growth. Traditional methods for evaluating optimal planting density primarily rely on agronomic traits, yield, and quality indicators, which suffer from significant lag and provide only superficial insights.

**Methods:**

In this study, the electrophysiological information was regarded as key indicator, and the influence of plant density on the growth of *P. heterophylla* was evaluated through multi-dimensional data.

**Results:**

The results indicate that there were significant differences (*p* < 0.05) in electrophysiological indices, electrical signals, intracellular water metabolism, nutrient transport and agronomic traits of *P. heterophylla* under different planting densities. Electrophysiological responses were faster and more sensitive than traditional methods, showing a non-linear pattern of “low in the middle and high at both ends”. The sensitivity of this response is reflected in the changes of growth state under density stress. When plant density was 80,000 to 100,000 plants per 667 m^2^, the above-ground part and underground part grew best, and total biomass and economic benefits were also optimal. When it was 100,000 plants per 667 m^2^, the saponin content reached 3.95%. Correlation analysis showed that the electrophysiological signal was positively correlated with the traditional evaluation index, and grey relational analysis indicated that each index at this density performed evenly and excellently. The change of electrophysiological signals had a high correlation with the results of grey relational analysis, which verified that electrophysiological information is an index for early, rapid, non-destructive indication of *P. heterophylla* growth status.

**Conclusion:**

This study demonstrates that electrophysiological information can serve as an early, rapid, non-destructive indicator of *P. heterophylla* growth status, and identifies 80,000–100,000 plants per 667 m^2^ as the optimal planting density. These findings provide a scientific basis for standardized cultivation of *P. heterophylla*.

## Graphical Abstract

**Figure:**
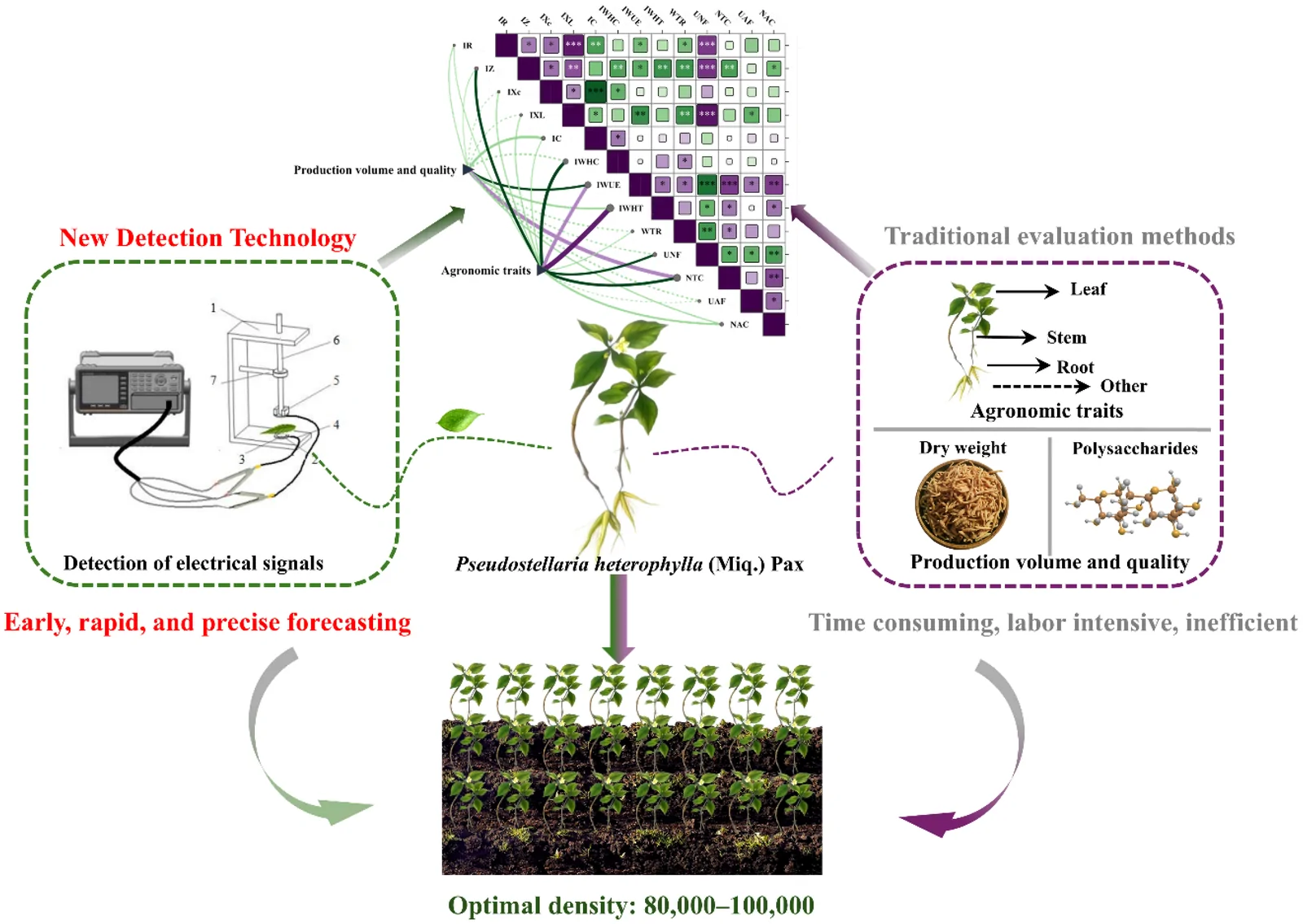

## Highlights

- This study provides the first evidence that electrophysiological signals can be used for early, rapid, and non-destructive detection of the effects of density stress on the *P. heterophylla.*
- The optimal planting density for *P. heterophylla* is 80,000 to 120,000 plants per 667 m².

## 1 Introduction

*Pseudostellaria heterophylla*, belonging to the family Caryophyllaceae, is the dried tuberous root of the plant known as ‘child ginseng’. It is a dried tuberous root that serves both as food and medicine (Song et al., 2025). Research indicates that extracts of *P. heterophylla* are rich in diverse active constituents including polysaccharides, saponins, cyclic peptides, and essential mineral elements. Approximately 289 phytochemicals have been isolated and identified from the dry roots (Li et al., 2022*;* Ng et al., 2022; Sha et al., 2023). Pharmacological studies show that *P. heterophylla* inhibits tumor growth (Gui et al., 2025), enhances memory function (He et al., 2024), alleviates inflammatory responses (Gao, 2021; You et al., 2021), modulates immunity (Choi et al., 2017; Liu et al., 2024), and lowers blood lipid and glucose levels (Chen et al., 2025; Liu et al., 2022). In clinical practice, it is primarily employed to treat fatigue and loss of appetite arising from spleen deficiency syndrome, whilst also alleviating dry cough symptoms caused by lung dryness (Cai et al., 2025; Lv and Wang, 2025). In addition, because it has nourishing properties, it is included in the “List of Items That Are Both Foods and Medicines” of the National Health Commission, and is also used in health preservation such as tea drinking and soup making (Liao and Tzen, 2022). On this basis, *P. heterophylla* has a unique position in both medicine and food, and also has various biological activities. Its medicinal value is relatively important, and its application prospects are relatively broad.

*P. heterophylla*, similar to those in Shibing, Zherong, Kuandian, is widely cultivated and distributed in China (Du et al., 2025; Kang et al., 2016b). Guizhou is a place with suitable climate in China, and the planting area of *P. heterophylla* is still relatively large (Kang et al., 2016a). However, the sustainable development of the local *P. heterophylla* industry still faces huge challenges. Planters find it difficult to control the structure of traditional cultivated populations, and unreasonable planting densities have long existed, which limits the simultaneous improvement of yield and quality. Planting density is an important matter in agriculture, which can have an impact on crop yield and quality, etc (Cao et al., 2022; Postma et al., 2021). Previous studies have shown that the most suitable planting density depends on the crop and is affected by the species and environmental conditions (Niharika et al., 2021). An ideal density and spatially uniform plant population are the basic prerequisite for efficient crop production and yield (García et al., 2025; Postma et al., 2021; Wilke et al., 2021).

In tradition, the growth, development and production performance evaluation of plants rely on agronomic trait investigation, yield measurement and quality analysis (Huang et al., 2025). It should be said that these methods often take both time and a lot of labor, and the yield or quality evaluation is only carried out after the plants mature and are harvested. Specifically for *P. heterophylla*, the growth cycle from tuber formation to harvest lasts 6 to 7 months (Yuan et al., 2021), and the content and composition of its active constituents—such as polysaccharides and cyclic peptides—are the core criteria for assessing the medicinal material’s quality. The traditional method needs to wait until the crop is harvested to evaluate the efficacy. The single-round breeding or cultivation test causes the cultivation experiment cycle to be extended to more than 1 year. There is a particularly urgent need for a method that can early identify the growth and development status of *P. heterophylla*, so as to promptly intervene to help its growth and increase production.

Electrophysiology is a dynamic and sensitive technique for measuring and controlling the electrical activity of cells, tissues, and organisms (Gao et al., 2022). Its application in plants has a relatively long history. The research history of plant electrophysiology can be traced back to 1873, when Burdon first observed the electrical signal transmission in the hairs of the Venus flytrap. This discovery marked the beginning of plant bioelectrophysiology research. Virtually all life processes in plants involve electrophysiological activity, encompassing charge separation, electron flow, and the transport of dielectric materials (Hedrich et al., 2016; Nguyen et al., 2018; Szechyńska et al., 2017). Electrical signals in plants are regarded as the most rapid response to abiotic or biotic stimuli (disease, drought, pests, salinity, cold, etc.) (Ai et al., 2025; Favre et al., 2011; Gallé et al., 2015). Zhang et al. (2020, 2021b, 2021c) pioneered the revelation within plant physiology of the intrinsic relationships and fitting equations between leaf resistance (R), impedance (Z), capacitive reactance (Xc), inductive reactance (XL), and clamping force (F). According to bioenergetic theory, the relationship between the aforementioned electrical parameters and clamping force can be uniformly expressed as y + ke^(-bF). When F = 0 (without the application of external force, in its natural state) and e^(0) = 1, the intrinsic R, Z, Xc and XL can be simplified to IR, IZ, IXc or IXL= *y*+*k*. Therefore, the capacitance (IC) can be directly calculated from IXc. This relationship demonstrates that these intrinsic electrical parameters can be utilized for real-time, rapid, accurate, and non-invasive physiological state monitoring. Moreover, extensive research has demonstrated that these electrical parameters are frequently employed to assess the physiological state of various plant species (Tian et al., 2024; Wei et al., 2024a; Yu et al., 2021; Zhang et al., 2021b). Research by Ali et al. (2022) showed that indicators such as R, Z, XL, and Xc reflected the nutrient absorption situation of mangroves, which helped to evaluate salt tolerance and provide reliable physiological information. Therefore, it is necessary to study whether plant electrophysiological information can reflect the impact of planting density on the growth of *P. heterophylla*, and then screen out the suitable planting density that can increase the yield.

To address the critical issue of low yields and unsustainable industrial development in *P. heterophylla* due to inappropriate planting densities, this study conducted a comprehensive evaluation of *P. heterophylla* under varying planting densities. This study for the first time systematically evaluated the electrophysiological responses of *P. heterophylla* under different planting densities, and explored its electrophysiological characteristics. The study used methods such as Comprehensive Growth Index (CGI) and gray correlation degree to comprehensively evaluate the optimal planting density of *P. heterophylla*. The purpose is to fully figure out the physiological and electrophysiological characteristics of *P. heterophylla*. The research on *P. heterophylla* was carried out under different planting densities, and a system for rapid non-destructive monitoring and evaluation was constructed. In order to determine the optimal planting density, improve its agronomic traits, and achieve the goal of high-yield and high-quality agriculture.

## 2 Materials and methods

### 2.1 Experimental site and materials

The *P. heterophylla*. samples used in this study belonged to the cultivar “Gui Shen No. 1,” sourced from the China Resources Jiangzhong *P. heterophylla* Standardized Cultivation Base in Niuchang Town, Shibing County, Qiandongnan Miao and Dong Autonomous Prefecture, Guizhou Province. The geographical coordinates of the region extend from 107°52′37″ to 108°28′47″ E longitude and 26°46′46″ to 27°20′16″ N latitude. This area is characterized by a warm, humid subtropical monsoon climate.

### 2.2 Experimental design

Field trials were conducted over the period from April to November 2024. The experiment utilized a randomized block design with five treatment groups, each replicated three times, totaling 15 independent plots. Each plot measured approximately 16 square meters in area. The specific layout is presented in **Table 1** and **Figure 1**.

**Table 1:** Field trial design plan.

Planting density(plants per 667 m^2^)	Plant spacing	Number of trees in the residential area
60,000	10 cm × 8 cm	1,280 plants per plot (32 plants × 10 rows × 4 beds)
80,000	7 cm × 8 cm	1,840 plants per plot (46 plants × 10 rows × 4 beds)
100,000	6 cm × 8 cm	2,160 plants per plot (54 plants × 10 rows × 4 beds)
120,000	5 cm × 8 cm	2,600 plants per plot (65 plants × 10 rows × 4 beds)
150,000	4 cm × 8 cm	3,240 plants per plot (81 plants × 10 rows × 4 beds)

1 mu = 667 m^2^. All planting densities are reported as plants per 667 m^2^ throughout the manuscript.

**Figure 1:**
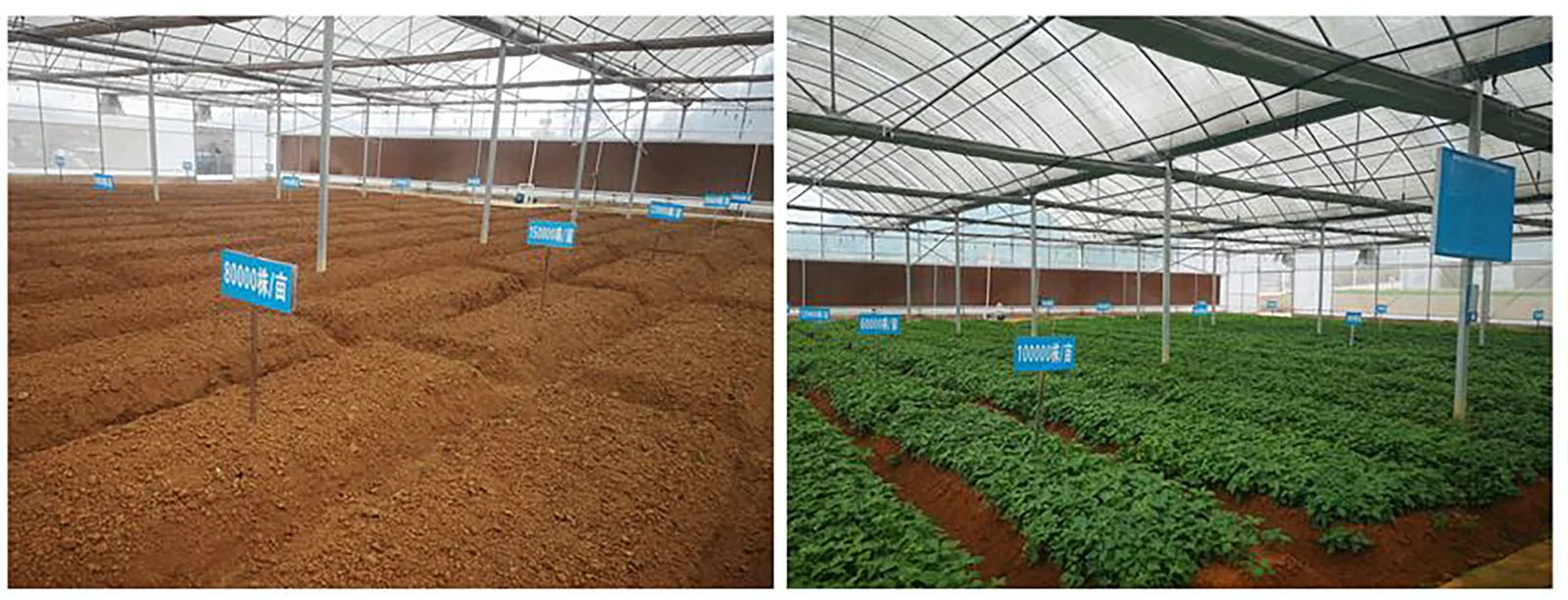
Field trial layout diagram.

### 2.3 Measurement of electrophysiological information

During the peak growth period (21 June 2024), fresh and fully expanded leaves of *P. heterophylla* were randomly selected from plots at different planting densities, harvested, and clearly labelled. Following the methodology described by Zhang et al. (2021b; 2021c), an LCR meter (LCR-6020) was used to apply a voltage of 1.5 V at a frequency of 3.0 kHz. Measurements of R, Z, XL, and Xc were taken at different clamping forces (1.139, 2.149, 3.178, 4.212, and 5.245 N). The corresponding parameters were then obtained from their respective fitting equations. When F = 0(without the application of external force, i.e., in its natural state), e^(0)=1; then R, Z, XL, and Xc were calculated according to Equation 1:

(1) IR, IZ, IXc or IXL=y+k

Subsequently, Equation 2 could be used to calculate IC:

(2) $$\text{IC}=\frac{1}{2\text{π}f_{\text{ IXc}}}$$

Where π = 3.1416, and *f* is the frequency.

Furthermore, the aforementioned five parameters enable the derivation of additional metrics for the leaves of *P. heterophylla*, including the intracellular water-holding capacity (IWHC), intracellular water utilization efficiency (IWHE), intracellular water holding time (IWHT), water or nutrient transfer rate (WTR or NTR), nutrient flux per unit area (UNF), nutrient transport capacity (NTC), nutrient active transport flux (NAF), nutrient active transport capacity (NAC), metabolic flux (MF), metabolic rate (MR), and metabolic activity (MA) (Zhang et al., 2025). The following Equations 3–14 are used to calculate these metrics:

(3) $$\text{IWHC}=\sqrt{{\left(\text{IC}\right)}^{3}}$$

(4) $$\text{IWHE}=\frac{\text{d}}{\text{IWHC}}$$

Where d represents the specific effective thickness of the plant leaf (in mm). Following the bioenergetic framework established by Zhang et al. (2020), d is derived from the linear relationship between leaf capacitance (C) and clamping force (F): $\text{C}={\text{X}}_{0}+\text{hF}$, where h is the slope of the linear regression. The specific effective thickness is then calculated as d = $\frac{{\text{U}}^{2}\text{h}}{2}$, where U is the test voltage (1.5 V). For each leaf, capacitance was measured at five clamping forces (1.139, 2.149, 3.178, 4.212, and 5.245 N), and the slope h was obtained from linear regression.

(5) IWHT=IC×IZ

(6) $$\text{WTR}\;\text{or}\;\text{NTR}=\frac{\text{IWHC}}{\text{IWHT}}$$

(7) $$\text{UNF}=\frac{\text{IR}}{\text{IXc}}+\frac{\text{IR}}{\text{IXL}}$$

(8) $$\text{NAF}=\frac{\text{IXc}}{\text{IXL}}$$

(9) NTC=NAF×NTR

(10) NAC=NAF×WTR

(11) $$\text{MF}=\frac{1}{\text{IR}\times \text{IZ}\times \text{IXc}\times \text{IXL}}$$

(12) MR=NTR×NAC 

(13) $$\text{MA}=\sqrt[{6}]{\text{MF}\times \text{MR}}$$

Moreover, we normalized and integrated key indicators (IWHC, IWHT, NTC, NAC, MA) to define the comprehensive growth index (CGI) of *P. heterophylla* as follows:

(14) $$\text{CGI}=\sqrt[{5}]{\text{IWHC}\times \text{IWHT}\times \text{NTC}\times \text{NAC}\times \text{MA}}$$

The five parameters selected for the CGI calculation, IWHC, IWHT, NTC, NAC, and MA, were chosen because they collectively represent two fundamental physiological processes that determine plant growth and stress tolerance: intracellular water metabolism and nutrient transport capacity (Zhang et al., 2025). Specifically, IWHC and IWHT reflect the capacity of cells to retain water and the duration of water retention, which are critical for maintaining turgor and metabolic activity under varying density conditions (Zhang et al., 2021c). NTC and NAC represent the efficiency of nutrient uptake and translocation, which directly influence biomass accumulation and yield formation (Wei et al., 2024b; Yu et al., 2021). MA serves as an integrative indicator of overall cellular metabolic vitality (Tian et al., 2024). Notably, although these five indicators are expressed in different units, they are all dimensionless in practical application because each is a relative ratio derived from the same set of intrinsic electrical parameters. The geometric mean was adopted to balance the contributions of all five parameters equally, without allowing any single parameter to dominate the composite index, and to ensure sensitivity to proportional changes across all dimensions. A similar multi-parameter integration approach using the geometric mean has been applied in previous studies on plant electrophysiological evaluation (Zhang et al., 2025; Tian et al., 2024; Wei et al., 2024b).

### 2.4 Assessment of agronomic traits

Ten randomly selected *P. heterophylla* plants from plots cultivated at different densities were assessed for agronomic traits. The parameters measured included leaf area, the number of leaves and branches, root and daughter root characteristics (weight, number, and diameter), as well as aboveground and underground traits (stem length, node number, and biomass). Fresh weight and dry weight of the plants were recorded and subsequently used to calculate yield per 667 m^2^. All agronomic trait indicators are detailed in **Table 2**.

**Table 2:** Agronomic traits measured in *P. heterophylla* and their corresponding measurement methods.

Agronomic traits	Measurement methods
Number of main stem branches	Number of stems and branches emerging from the root collar.
Number of primary branches	Number of primary branches on the main stem.
Number of leaves	Total number of unfolded leaves per plant.
Number of underground stem nodes	Number of stem nodes from the stem base to the ground.
Number of aboveground stem nodes	Number of nodes from base to apex.
Number of daughter roots	Adventitious roots, including rhizomes and adventitious roots at the base of the rootstock.
Number of expanded daughter roots	Number of tuberous roots, i.e., adventitious roots swollen into tubers.
Total plant length	Length from root tip to stem top.
Underground stem length	Distance from the stem base to the ground, including incomplete stem segments.
Aboveground stem length	Above-ground stem length, including incomplete stem segments.
Leaf area	The area of a plant leaf’s single surface.
Diameter of expanded daughter roots	Mean of the maximum diameters of three randomly selected tuberous roots.
Stem diameter	The diameter of the first above-ground stem node emerging from the most vigorous shoot growing from the root crown base.
Diameter of expanded daughter roots Weight	Fresh weight of enlarged lateral roots.
Total biomass	The fresh weight of the plant’s aboveground and belowground parts, i.e., the entire plant.
Underground biomass	Fresh weight of all belowground parts, including stems, roots, and tubers.
Aboveground biomass	Fresh weight of aboveground stems, leaves, and flowers.

Furthermore, the calculation of leaf area is given by Equation 15:

(15) Leaf area=0.666×leaf length×leaf width

### 2.5 Determination of yield and quality

During the harvesting period, underground tuberous organs of *P. heterophylla* were systematically excavated from all trial plots. The specific steps are as follows: a. Excavate a 1.0 m² area at the center of the community to collect rhizome samples of *P. heterophylla*. b. Carefully remove impurities and wash the sample, then allow the surface moisture of the tuberous roots to air-dry. c. Weigh the fresh sample, then dry it at 55 °C until constant weight is achieved, after which the dry weight was determined. d. Calculate the dry matter conversion factor based on the ratio of dry to fresh weight, and use this to convert the yield per 667 m^2^.

Furthermore, the moisture and ash content of *P. heterophylla* were determined using the oven-drying method and muffle furnace (Lee et al., 2024; Sharma et al., 2024). The composition of leachate was quantitatively analyzed by using the method of Wang et al. (2022) for cold water extraction: extraction with water, then separation of polysaccharides by ethanol precipitation, and finally determination of the content by the phenol-sulfuric acid method. The determination of saponin content was performed using the vanillin-sulphate colorimetric method established by Birhanie et al. (2022).

### 2.6 Data processing and analysis

Microsoft Office LTSC Professional Plus 2024 was used for data processing and aggregation. The relationship between electrophysiological parameters and clamping force was fitted using SigmaPlot 12.5. For agronomic trait measurements, 10 plants were randomly sampled from each plot, and the measurements were averaged by plot prior to statistical analysis. The plot (approximately 16 m²), rather than individual plants or leaves, was considered the experimental unit (n = 3 plots per treatment). Statistical analyses were performed using one-way analysis of variance (ANOVA) with planting density as a fixed factor. As recommended by Hu and Hu (2023), in randomized complete block designs where block effects are not the primary focus and the experimental field is relatively homogeneous, one-way ANOVA with treatment as the main factor is a valid analytical approach. Tukey’s HSD test was used for *post-hoc* pairwise comparisons. All values are presented as plot-level means ± standard deviation. Charts and tables were created using GraphPad Prism 10.1 and PowerPoint.

The correlation heatmap and Mantel test in this study were produced with the online tool ChiPlot (https://www.chiplot.online/), which implements the linkET R package for these calculations. The distance matrix for electrophysiological parameters was calculated using Euclidean distance, while the matrix for agronomic, yield, and quality traits was calculated using Gower’s distance. Significance was assessed based on 9,999 permutations. The correlations between distance matrices were visualized with line width representing Mantel’s “r” statistic and line color representing Mantel’s “*p*” statistic.

Evaluate *P. heterophylla* under different densities using the grey relational degree coefficient. Experimental data were normalized with reference to a sequence denoted as $X_{i}\;$(i=0, 1, 2…, 37). The optimal value of each index was selected as the reference sequence $X_{0}$(*k*), and the distinguishing coefficient is 0.05, where *k =* {1, 2, 3…, m}. Equation 16 was used to calculate the correlation coefficient S for each sequence and the reference sequence (Tian et al., 2025b):

(16) $$S_{i}=\frac{{min}_{i}{min}_{k\;}\left|{\text{x}}_{0}\left(k\right)-{\text{x}}_{\text{i}}\left(k\right)\right|\;+\;\rho \;\cdot \;{m\text{a}\text{x}}_{i}{max}_{k\;}\left|{\text{x}}_{0}\left(k\right)-{\text{x}}_{\text{i}}\left(k\right)\right|}{\left|{\text{x}}_{0}\left(k\right)-{\text{x}}_{\text{i}}\left(k\right)\right|+\rho \;\cdot \;{max}_{i}{max}_{k\;}\left|{\text{x}}_{0}\left(k\right)-{\text{x}}_{\text{i}}\left(k\right)\right|}$$

The Entropy Weighting Method (EWM) is employed to determine the weights (*W*) for each evaluation criterion. The weighted correlation coefficient (*R*) is calculated using Equation 17 as follows:

(17) $$R_{0i}=\frac{1}{m}\sum_{\text{k}=1}^{\text{m}}W_{k}\cdot S\left(k\right)$$

## 3 Results

### 3.1 Effects of different planting densities on the electrophysiological information of *P. heterophylla*

The effects of different cultivation densities on the electrophysiology of *P. heterophylla* are shown in **Table 3**. The IC of *P. heterophylla* ranged from 43.50 to 57.79 pF at planting densities between 60,000 and 120,000 plants per 667 m^2^ However, it decreased to 32.40 pF at a higher density of 150,000 plants per 667 m^2^. The planting density of 150,000 plants per 667 m^2^ represented a 47.4% reduction (*p* < 0.05) compared to the previous density level of 120,000 plants per 667 m^2^. The IR, IZ, IXL, and IXc values of *P. heterophylla* collectively showed a non-linear response pattern characterized by “lower values in the middle and higher values at both ends”. At a planting density of 60,000 plants per 667 m^2^, the aforementioned parameters for *P. heterophylla* were recorded at elevated levels (4.79 MΩ, 1.17 MΩ, 5.22 MΩ, 1.23 MΩ). However, at planting densities of 80,000 to 100,000 plants per 667 m^2^, the overall trend declined, with a slight recovery observed at a density of 120,000 plants per 667 m^2^. But it decreased to 32.40 pF at a higher density of 150,000 plants per 667 m^2^, IC exhibited the lowest values, while IR, IZ, IXL, and IXc showed the highest. Compared to a density of 120,000 plants per 667 m^2^, the increases reached 141.3%, 18.6%, 70.2%, and 92.5% respectively.

**Table 3:** Effects of different planting densities on IC, IR, IZ, IXL, and IXc in *P. heterophylla*.

Planting density (plants per 667 m^2^)	IC (pF)	IR (MΩ)	IZ (MΩ)	IXL (MΩ)	IXc (MΩ)
60000	43.50 ± 3.24 ab	4.79 ± 0.80 ab	1.17 ± 0.09 b	5.22 ± 1.28 b	1.23 ± 0.09 b
80000	54.40 ± 4.80 a	2.35 ± 0.70 b	1.06 ± 0.11 b	2.78 ± 0.87 b	0.98 ± 0.09 b
100000	49.84 ± 3.92 a	4.34 ± 0.98 ab	0.99 ± 0.13 b	4.86 ± 1.10 b	1.07 ± 0.08 b
120000	57.79 ± 5.34 a	2.83 ± 0.48 b	1.29 ± 0.09 ab	5.03 ± 1.18 b	0.93 ± 0.09 b
150000	30.40 ± 4.82 b	6.83 ± 1.17 a	1.53 ± 0.08 a	8.86 ± 1.04 a	1.79 ± 0.29 a
*F* values	11.599	8.484	8.674	7.943	10.778
*p* values	< 0.001	0.003	0.003	0.004	0.001

IC, IR, IZ, IXL, and IXc represent intrinsic capacitance, intrinsic resistance, intrinsic impedance, intrinsic inductive resistance, and intrinsic capacitive resistance, respectively. At the statistical level, when the *p* value is less than 0.01, the differences between the groups represented by different lowercase letters observed are highly significant. The standard deviation for three biological replicates (n = 3).

**Figures 2**, **3** reveal that during the vigorous growth phase, planting density significantly influences the water metabolism and nutrient transport processes within the cells of *P. heterophylla*. For *P. heterophylla*, no significant differences were observed in IWHC and WTR/NTR among treatments ranging from 60,000 to 120,000 plants per 667 m^2^. However, at a density of 150,000 plants per 667 m^2^, a marked reduction was observed, which was significantly different from other treatment groups (*p* < 0.05). Conversely, the IWUE of *P. heterophylla* was highest at a planting density of 150,000 plants per 667 m^2^ and was significantly higher than in other treatment groups (*p* < 0.001). IWHT reached the highest value at 120,000 plants per 667 m^2^ and the lowest value at 150,000 plants per 667 m^2^. In this study, no difference in UNF under different density was found. The overall trends of NTC, NAF, and NAC of *P. heterophylla* were consistent with the electrophysiological signals, showing a non-linear characteristic of “low in the middle and high at both ends”. At 150,000 plants per 667 m^2^, this trend continued to develop downward.

**Figure 2:**
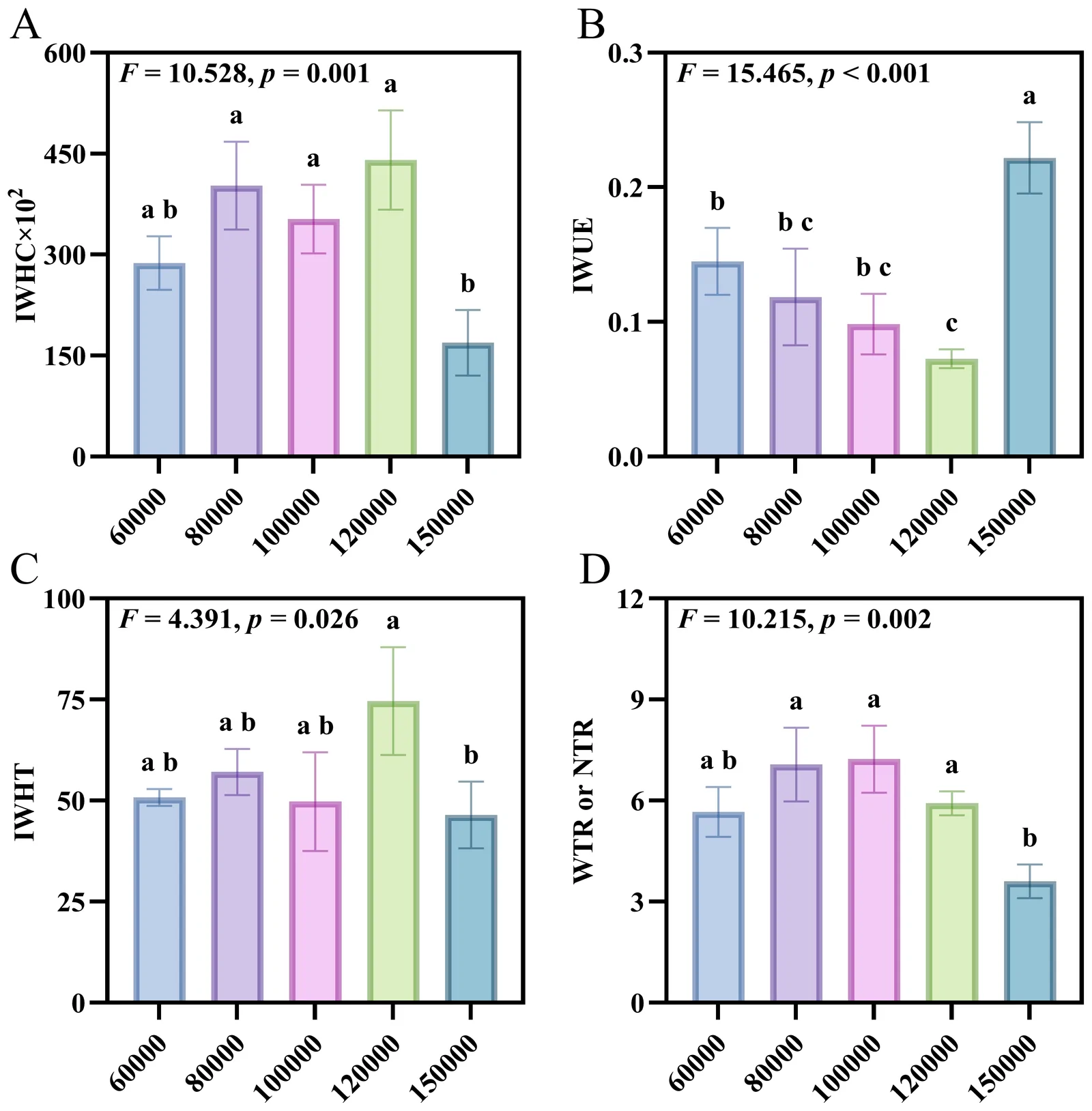
Effects of planting density on cellular water metabolism in *P. heterophylla.* **(A)** Intracellular water-holding capacity (IWHC), **(B)** Intracellular water utilization efficiency (IWUE), **(C)** Intracellular water-holding time (IWHT), **(D)** Water or nutrient transfer rate (WTR or NTR). The bar chart with error bars presents the means and standard deviations of three replicate plots per treatment (n = 3). Through Tukey test analysis, the different letter marks in the figure represent statistically significant differences (*p* < 0.05) among the measured indices.

**Figure 3:**
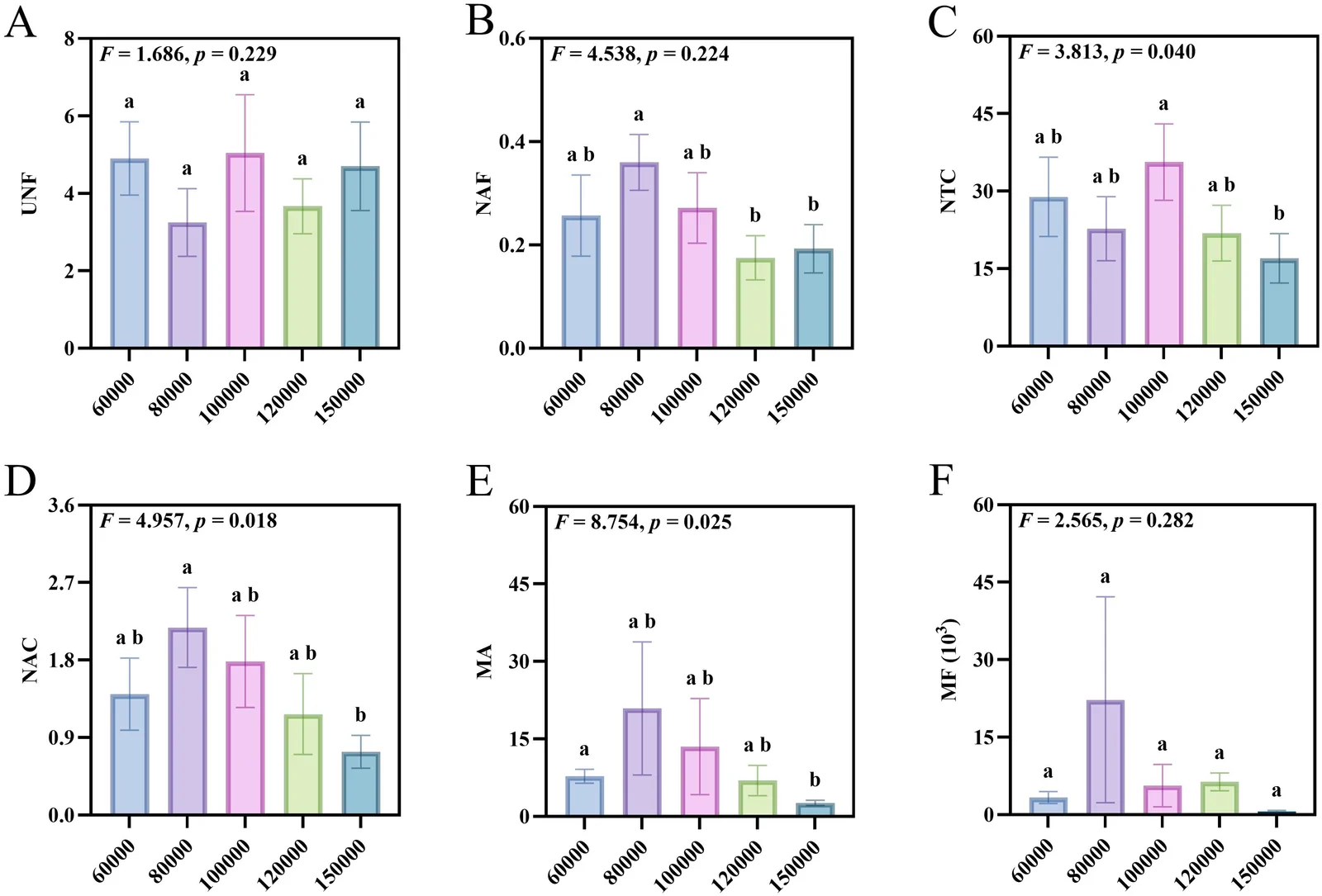
Effects of Planting Density on Intracellular Nutrient Translocation in *P. heterophylla.* **(A)** Nutrient flux per unit area (UNF), **(B)** The active transport flow of nutrient (NAF), **(C)** Nutrient transport capacity (NTC), **(D)** Nutrient active transport capacity (NAC), **(E)** Metabolic activity (MA), **(F)** Metabolic flow (MF). The bar chart with error bars presents the means and standard deviations of three replicate plots per treatment (n = 3). Through Tukey test analysis, the different letter marks in the figure represent statistically significant differences (*p* < 0.05) among the measured indices.

### 3.2 Effects of planting density on agronomic traits of *P. heterophylla*

As shown in **Figures 4** and **5**, under different cultivation densities, the aboveground and belowground agronomic traits of *P. heterophylla* exhibited significant differences. In terms of aboveground biomass, the 100,000 plants per 667 m^2^ treatment resulted in the greatest total plant length at 26.93 ± 0.81 cm, whereas the 150,000 plants per 667 m^2^ treatment produced the smallest at 20.47 ± 1.15 cm. In terms of leaf area, the leaf area was the largest when there were 60,000 plants per 667 m^2^, which had strong photosynthetic potential. While the leaf area was the smallest when there were 150,000 plants per 667 m^2^, maybe because high density inhibited the expansion of leaves. The overall average of stem diameter was about 0.21 cm, and the stem diameter was the largest when there were 100,000 plants per 667 m^2^, which was 0.27 cm. Under different treatment conditions, the number of main stems was between 1 and 6. When planting 100,000 plants per 667 m^2^, the number of main stems was more than that of other treatments, and when planting 80,000 plants per 667 m^2^, the number of main stems ranked second. The number of above - ground stem nodes was 2-9. The number of stem nodes was relatively small when planting 60,000 plants per 667 m^2^ and 120,000 plants per 667 m^2^. The above - ground biomass was the highest when planting 100,000 plants per 667 m^2^, and there was not much difference among other treatments.

**Figure 4:**
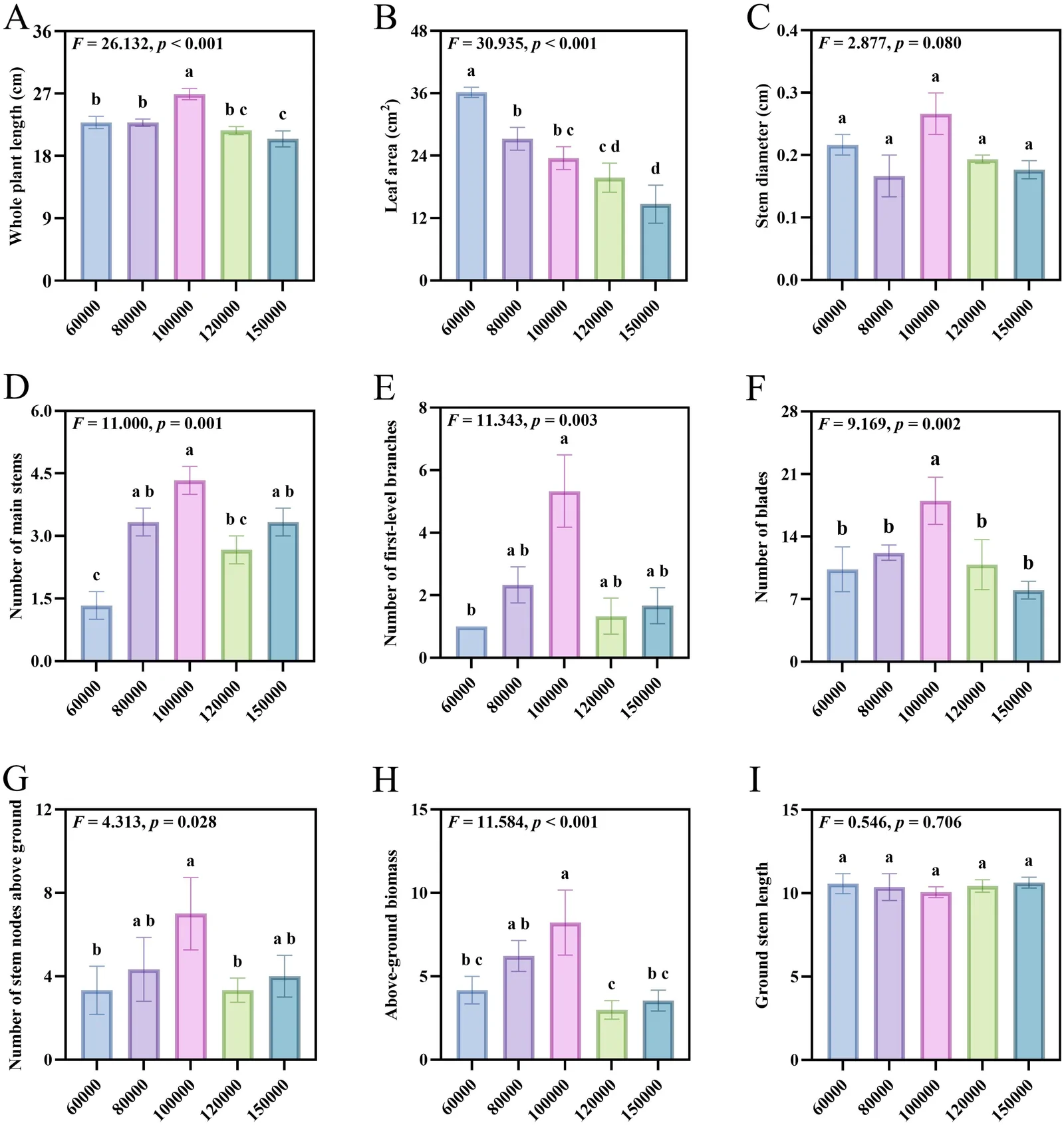
Effects of planting density on aboveground agronomic traits of *P. heterophylla.* **(A)** Whole plant length, **(B)** Leaf area, **(C)** Stem diameter, **(D)** Number of main stems, **(E)** Number of first-level branches, **(F)** Number of blades, **(G)** Number of stem nodes above ground, **(H)** Above-ground biomass, **(I)** Ground stem length. The bar chart with error bars presents the means and standard deviations of three replicate plots per treatment (n = 3). Through Tukey test analysis, the different letter marks in the figure represent statistically significant differences (*p* < 0.05) among the measured indices.

**Figure 5:**
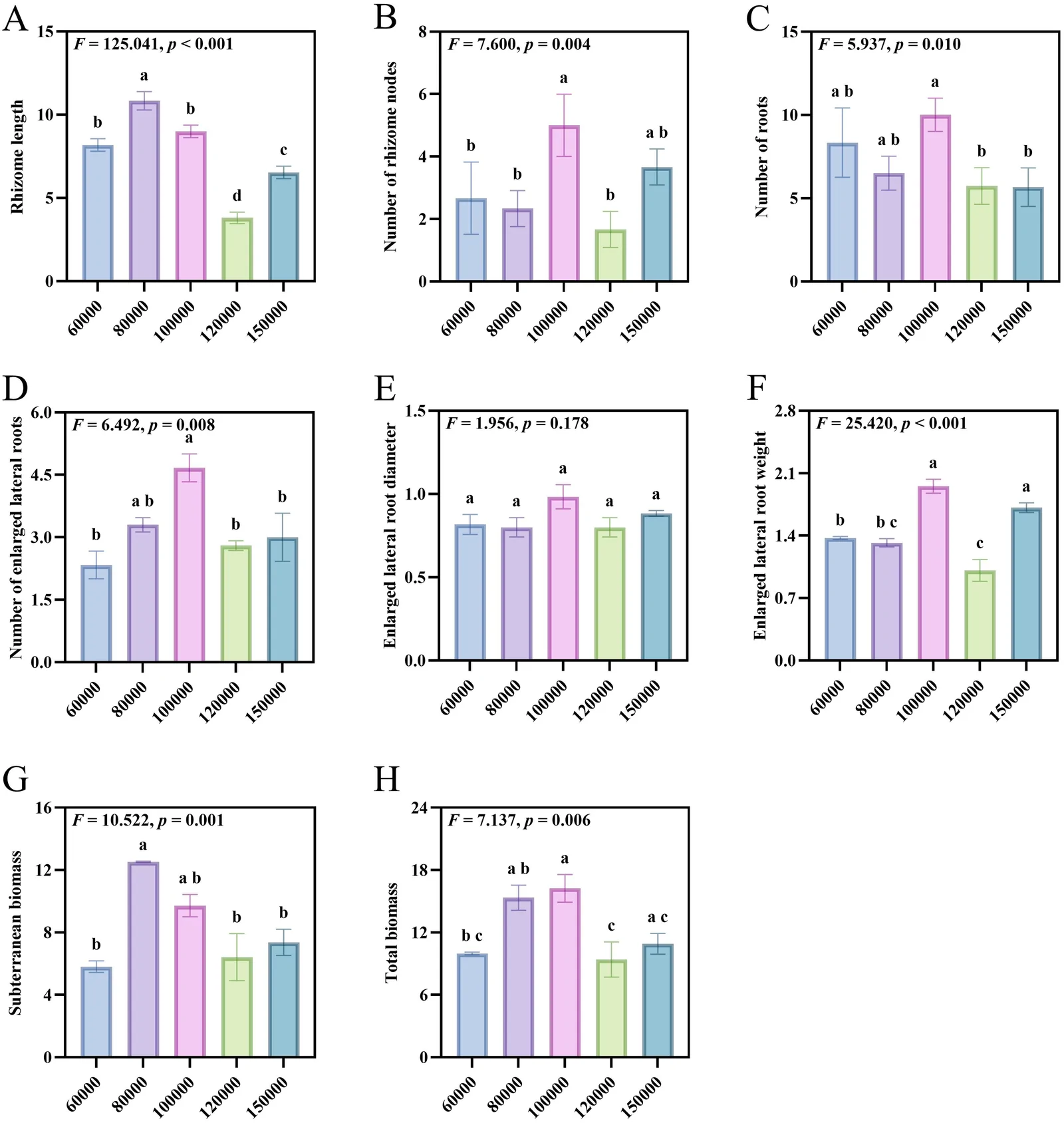
Effects of planting density on belowground agronomic traits of *P. heterophylla.* **(A)** Rhizome length, **(B)** Number of rhizome nodes, **(C)** Number of roots, **(D)** Number of enlarged lateral roots, **(E)** Enlarged lateral root diameter, **(F)** Enlarged lateral root weight, **(G)** Subterranean biomass, **(H)** Total biomass. The bar chart with error bars presents the means and standard deviations of three replicate plots per treatment (n = 3). Through Tukey test analysis, the different letter marks in the figure represent statistically significant differences (*p* < 0.05) among the measured indices.

The underground part character of *P. heterophylla* was related to yield and quality, and also affected the medicinal and economic situations. This was the key point in cultivation research and management. This study showed that different planting densities had obvious effects on the underground part character of *P. heterophylla*. As shown in **Figure 5**, there were differences in rhizome length under different treatments. When the number of plants per 667 m^2^ was 80,000, the longest rhizome appeared, and its length was 10.83 ± 0.55 cm; when the number of plants per 667 m^2^ was 120,000, the shortest rhizome appeared, and its length was 3.80 ± 0.35 cm. The number of rhizome nodes ranged from 1 to 6, with the highest number of rhizome nodes recorded at 100,000 plants per 667 m^2^. No significant differences were observed among other density treatments. The enlarged lateral roots of *P. heterophylla* measured between 0.70 and 1.10 cm in diameter, with the highest average diameter of 0.98 ± 0.13 cm observed in plants cultivated at a density of 100,000 plants per 667 m^2^. The weight of enlarged lateral roots also exhibited marked variation, ranging from 0.85 g to 2.01 g, with the heaviest recorded at 100,000 plants per 667 m^2^ and the lightest at 120,000 plants per 667 m^2^. There was a relationship between biomass and density. The above - ground biomass and total biomass at 80,000 plants per 667 m^2^ and 100,000 plants per 667 m^2^ were the highest, and there was no obvious difference in other densities.

### 3.3 Effects of planting density on yield and quality of *P. heterophylla*

*P. heterophylla* is a rhizomatous herbaceous plant. The yield reflects the economic value (Zhang et al., 2021d). The key of the economy lies in the dry weight, not the fresh weight. The fresh weight is the initially harvested sample, and it needs to be dried before it can become the root tuber for sale. In this study, the fresh weight and dry weight under the density of 60,000-150,000 plants per 667 m^2^ were measured, and the relationship between the yield formation of *P. heterophylla* and density was analyzed. As shown in **Figure 6**, the highest fresh weight yield per 667 m^2^ was achieved at 150,000 plants per 667 m^2^; however, fresh weight showed no direct correlation with economic returns. The lowest fresh weight yield occurred at 120,000 plants per 667 m^2^. The highest dry matter yield occurred when the plant density was 80,000 plants per 667 m^2^, and the lowest occurred when the plant density was 120,000 plants per 667 m^2^. The density of 150,000 plants per 667 m^2^ was higher, but the dry matter yield did not increase proportionally. This result indicates that the cost saved by simply increasing the density was not transformed into economic benefits, and might even have the opposite effect.

**Figure 6:**
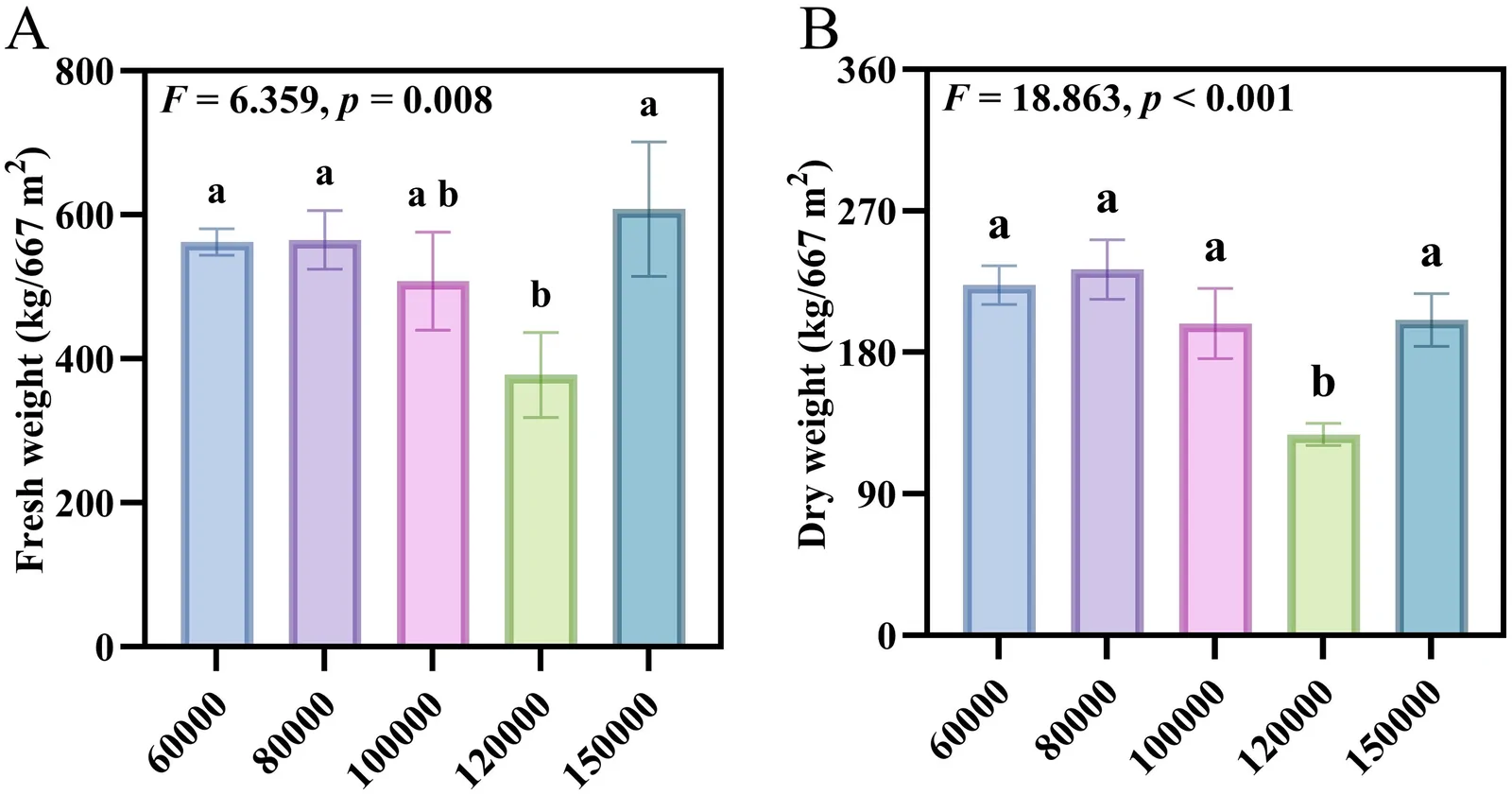
Yield variation of *P. heterophylla* at different planting densities. **(A)** Fresh weight, **(B)** Dry weight. The bar chart with error bars presents the means and standard deviations of three replicate plots per treatment (n = 3). Through Tukey test analysis, the different letter marks in the figure represent statistically significant differences (*p* < 0.05) among the measured indices.

The effects of different cultivation densities on the quality of *P. heterophylla* are shown in **Table 4**. Moisture content of *P. heterophylla* did not differ significantly across cultivation densities, with an average moisture content of 62.41%. The ash content was between 2.47% and 2.89%. The highest ash content was observed at a cultivation density of 120,000 plants per 667 m^2^ and the lowest was observed at 80,000 plants per 667 m^2^. The saponin and ash contents in the whole *P. heterophylla* plant were comparable, with an average value of 2.87%. However, the saponin content in *P. heterophylla* showed a significant difference in proportion (*p* < 0.05). At a cultivation density of 100,000 plants per 667 m^2^, it reached 3.95%, which is 1.7 percentage points higher than the lowest proportion observed at 80,000 plants per 667 m^2^. Polysaccharides, which are one of the important active components of *P. heterophylla*, are typically present in substantial quantities (Xie et al., 2023). The average polysaccharide content in *P. heterophylla* cultivated at different densities was 45.67%, ranging from 42.77% to 50.85%.

**Table 4:** Effects of different planting densities on the nutritional quality of *P. heterophylla*.

Planting density (plants per 667 m^2^)	Moisture content (%)	Ash content (%)	Saponin content (%)	Extractable content (%)	Polysaccharide content (%)
60000	60.40 ± 0.91 a	2.79 ± 0.03 a	2.42 ± 0.11 bc	38.12 ± 0.67 a	44.03 ± 1.49 bc
80000	58.53 ± 4.90 a	2.47 ± 0.10 ab	2.25 ± 0.20 c	36.55 ± 1.67 a	42.77 ± 1.51 c
100000	60.78 ± 3.18 a	2.58 ± 0.17 ab	3.95 ± 0.16 a	37.59 ± 2.21 a	43.28 ± 1.21 bc
120000	65.67 ± 3.88 a	2.89 ± 0.02 a	2.77 ± 0.12 bc	38.40 ± 0.56 a	47.40 ± 1.00 ab
150000	66.68 ± 2.90 a	2.59 ± 0.03 b	2.95 ± 0.20 b	38.44 ± 0.82 a	50.85 ± 1.59 a
*F* values	2.168	29.136	33.580	0.451	12.270
*p* values	0.146	< 0.001	< 0.001	0.770	< 0.001

Data are presented as the mean ± standard deviation of three biological replicates (n = 3). Through Tukey test analysis, the different letter marks in the figure represent statistically significant differences (*p* < 0.05) among the measured indices.

### 3.4 Correlation analysis

Changes in the electrophysiological parameters of *P. heterophylla* not only characterize the effects of different cultivation densities on yield and quality, but also reveal the early influence of cultivation densities on agronomic traits. Using Spearman’s and Mantel tests, a correlation matrix was established between electrophysiological signals and agronomic traits, yield and quality parameters. **Figure 7** presents the results of the Mantel test comparing electrophysiological signals to yield, quality, and agronomic traits of *P. heterophylla* cultivated at different densities. Yield and quality exhibited significant positive correlations (*p* < 0.05) with IWUE and NTC in the electrophysiological signals. Agronomic traits showed positive correlations with the electrophysiological signals IWHT, IWUE, IWHC, IZ, and NTC (*p* < 0.05). The Mantel test further confirmed that the electrophysiological signals of *P. heterophylla* could be used as an early indicator of the effects of planting density on yield, quality, and agronomic traits.

**Figure 7:**
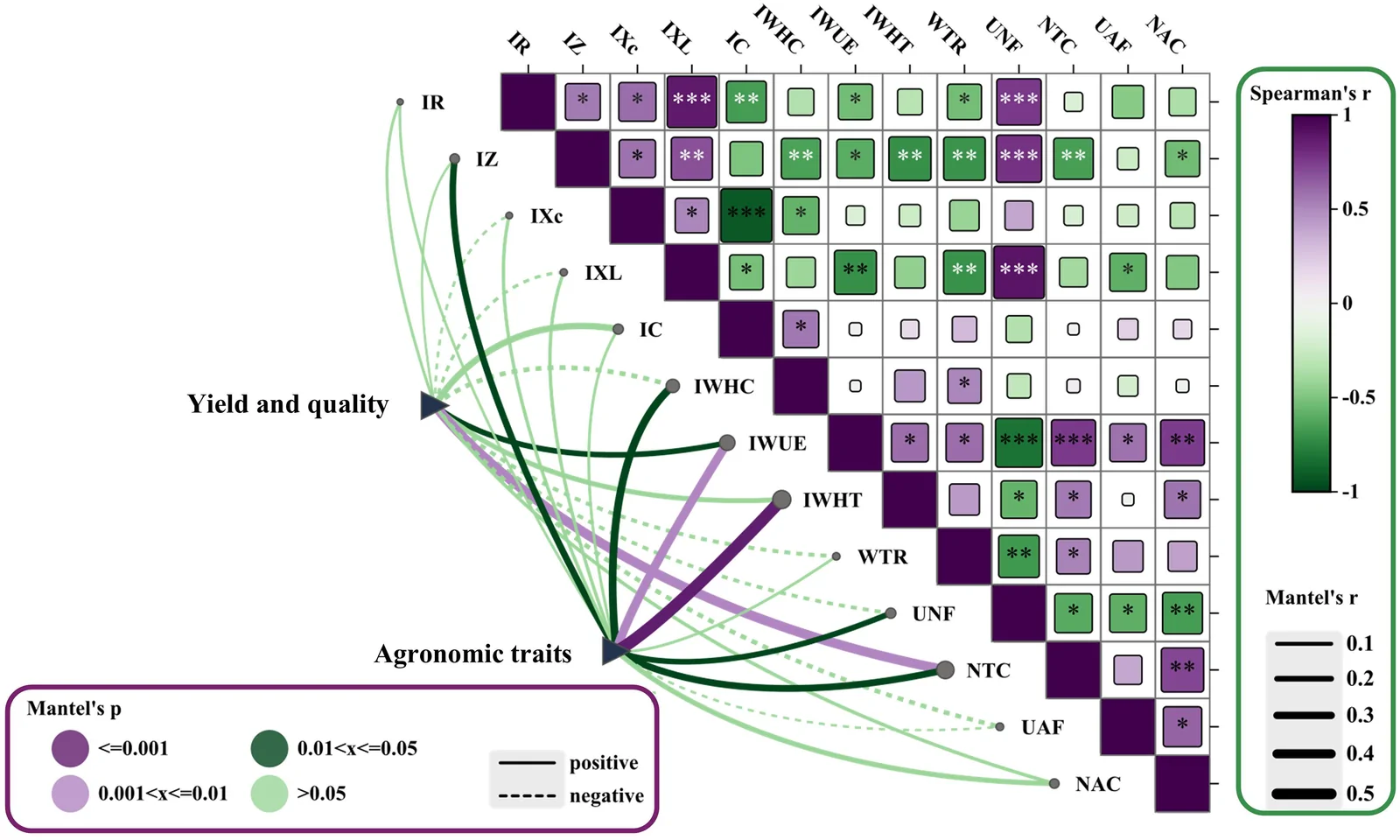
Correlation analysis of electrophysiological signals with agronomic traits, yield, and quality. Line width corresponds to Mantel’s r statistic; line color corresponds to Mantel’s *p* statistic; the color gradient represents Spearman’s r correlation coefficient for pairwise comparisons of *P. heterophylla* electrophysiological data.

### 3.5 Comprehensive evaluation of the effects of planting density on *P. heterophylla*

**Figure 8** presents the comprehensive evaluation of the vigor of the growth stage of *P. heterophylla* under different plant densities. There were significant differences in the CGI of *P. heterophylla* under different cultivation densities (*p* < 0.001, **Figure 8A**). In this study, the CGI of *P. heterophylla* reached the top when there were 80,000 plants per 667 m^2^ and was at the lowest when there were 150,000 plants per 667 m^2^. It should be noted that the difference in CGI between 80,000 plants per 667 m^2^ and 120,000 plants per 667 m^2^ was not obvious. Therefore, the density of 80,000 to 120,000 plants per 667 m^2^ belonged to the appropriate density for the growth of *P. heterophylla*. When there were less than 80,000 plants per 667 m^2^, the population was not vigorous; when there were more than 120,000 plants per 667 m^2^, density stress occurred. The CGI using plant sensor signals could reflect the effects of different planting densities on the growth and stress response of *P. heterophylla*.

**Figure 8:**
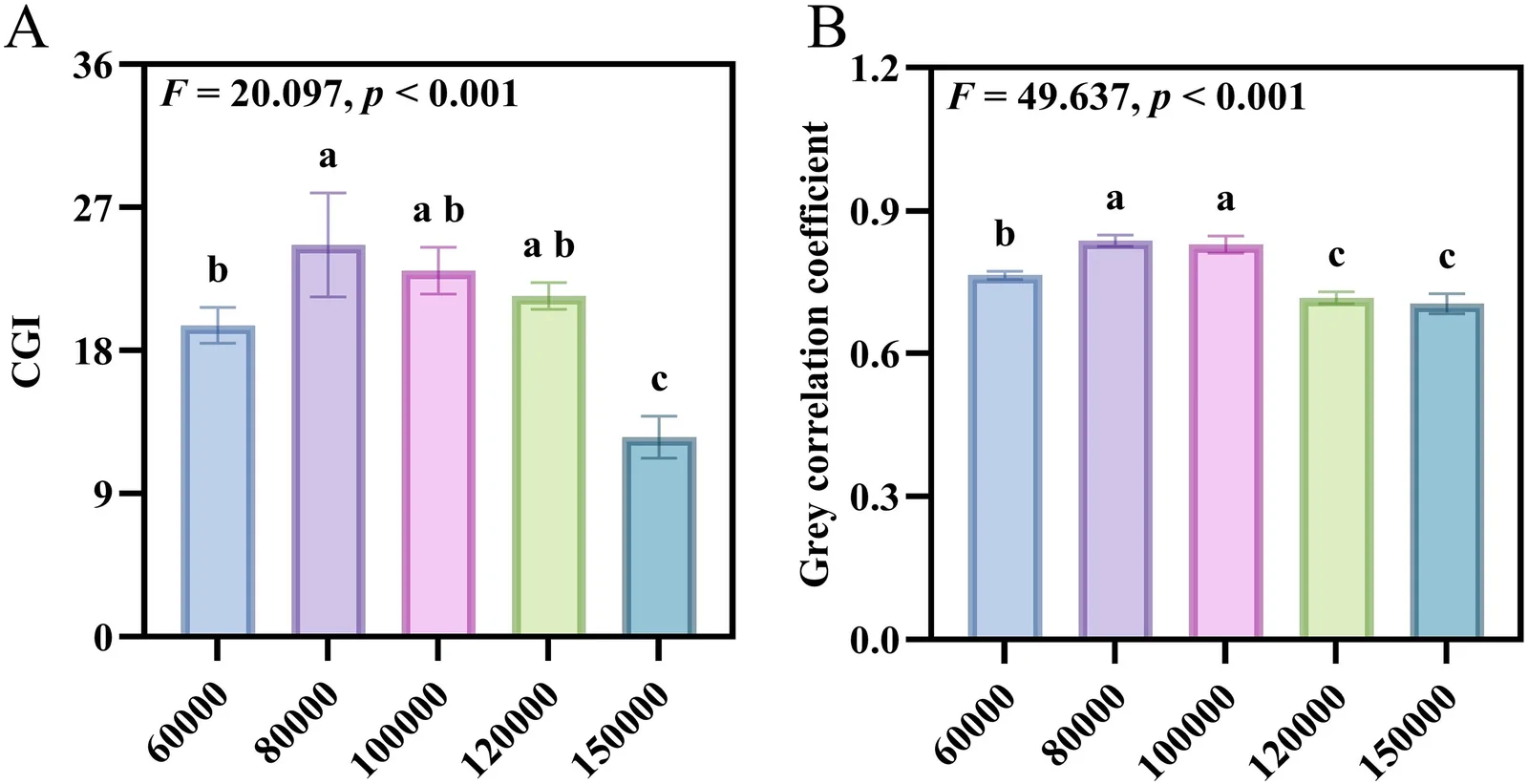
Comprehensive evaluation of the effects of different planting densities on *P. heterophylla.* **(A)** Composite growth index (CGI), **(B)** Grey correlation coefficient. The bar chart with error bars presents the means and standard deviations of three replicate plots per treatment (n = 3). Through Tukey test analysis, the different letter marks in the figure represent statistically significant differences (*p* < 0.05) among the measured indices.

The grey relational analysis method was used to connect the electrophysiological data and agronomic traits, and to evaluate the competitive performance of *P. heterophylla* under different density stress. The grey relational degrees between the growth parameters of *P. heterophylla* under different stress densities and the ideal reference sequence were calculated, and the results are shown in **Figure 8B**. In the case of Compr- eval, the comprehensive score of *P. heterophylla* at 80,000 plants per 667 m^2^ was the highest, and there was no significant difference compared with 100,000 plants per 667 m^2^ (*p* < 0.05). The evaluation results of CGI and gray relational grade were highly consistent.

## 4 Discussion

Plant electrophysiological signals can rapidly reflect changes in both internal growth conditions and external environmental factors, which are of significant physiological importance (Yu et al., 2021). Studies have shown that plants exhibiting robust growth typically display characteristics of high capacitance and low resistance (Nguyen et al., 2018; Szechyńska et al., 2017; Zhang et al., 2025). Conversely, when plants encounter stress conditions, the structural integrity of cell membranes is often compromised. This results in increased leakage of intracellular substances, leading to a decrease in IC and a corresponding increase in IR. This electrophysiological shift is typically regarded as an indicator of diminished vitality (Gao et al., 1997). Our previous research indicated that continuous cropping stress increases IR, IZ, IXL, and IXc in plants while reducing IC (Tian et al., 2024b; Wei et al., 2024b; Zhang et al., 2025).

Density stress is also one of the key factors influencing plant growth and development (Zhang et al., 2024b). In this study, significant variations in electrophysiological signals such as IC and IR were observed in *P. heterophylla* across different cultivation densities, which exhibited a non-linear response. The results showed that both extremely low and extremely high densities of *P. heterophylla* could damage the electrophysiological signals. When the density is between 80,000 and 120,000 plants per 667 m^2^, the electrophysiological signals are in a stable state, which confirms that the electrophysiological signals can reasonably reflect the growth changes of *P. heterophylla* related to density.

IWUE is an index of plant water-holding capacity, which is a key index reflecting the relationship between plant water consumption and accumulation (Feng et al., 2022). Under favorable circumstances, plants have a higher IWHC and a lower IWUE. In adverse conditions, the uneven distribution of soil moisture leads to an increase in IWUE, thus meeting their own needs (Sa, 2024; Zhang et al., 2021c). In recent years, more research has used the relevant contents of intracellular water metabolism and nutrient transport to evaluate the physiological states of Chinese broccoli, tomatoes, rapeseed, *P. heterophylla*, *Orychophragmus violaceus*, and *Pinellia ternata* (Tian et al., 2025a; Wei et al., 2024b; Xing et al., 2022; Zhang, 2021a, 2024a, 2025). Zhang et al. found that continuous planting for 1–4 years inhibited the intracellular water metabolism and nutrient transport of *Pinellia ternata*. The results of this study showed that different planting densities had different effects on the intracellular water metabolism and nutrient transport of *P. heterophylla*, and it was also confirmed that too high planting density would damage the growth of the plant.

The agronomic traits of crops belong to phenotypic characteristics, which are visible and associated with yield, and are the key to evaluating medicinal plant germplasm resources (Zhu et al., 2019). Low-density planting can optimize the agronomic traits of individual plants. High density may increase the leaf area index, but excessive crowding will lead to canopy closure, reduced air circulation and light penetration, and a significant decrease in photosynthetic efficiency (Khan et al., 2017; Wu et al., 2020). Planting density is an important factor affecting plant biomass. The biomass of plants increases with the increase in density (Yao et al., 2026). A larger leaf area may have more photosynthesis, which is beneficial for plant growth (Guo et al., 2024), but an overly large leaf area may lead to nutrient competition and a decrease in fruit quality (Wu et al., 2025). In this study, the leaf area of *P. heterophylla* at 60,000 plants per 667 m^2^ was the largest. But its underground biomass and total biomass were lower than those of *P. heterophylla* at 80,000 and 100,000 plants per 667 m^2^. This was in line with the description of the leaf area-photosynthesis relationship mentioned earlier.

Planting density significantly regulates the synergistic growth relationship between the aboveground and belowground parts of *P. heterophylla* by redistributing resources such as sunlight, water, and nutrients, thereby establishing a dynamic source-sink equilibrium. The mutual coordination between the quantity and functions of the two types of organelles in the source and sink tissues constitutes a crucial determinant of crop productivity (Li and Tu, 2002). The stems and leaves of *P. heterophylla* are used for photosynthesis and assimilation. The underground tuberous roots are sinks, used to accumulate photosynthetic assimilates and form economic yields (Deng et al., 2021; Zhang et al., 2019).

When planting 80,000-100,000 plants per 667 m^2^, there is no additional seedling cost, the dry weight is the highest, and it is most favorable for the balance of cost and benefit. When planting 150,000 plants per 667 m^2^, the fresh weight is higher, but the seedling and field management costs increase, the dry weight remains unchanged, and the economic benefit is not good. When planting 120,000 plants per 667 m^2^, the dry weight is lower, which would likely affect grower returns. However, a formal cost-benefit analysis incorporating seedling costs, field management, and market prices would be required to draw definitive conclusions on economic optimality.

Grey relational analysis is a key method in grey system theory, and it has been widely applied in multiple disciplines such as complex system analysis and multi-criteria evaluation in recent years (Jiang et al., 2025; Tian et al., 2025b). In this study, when 80,000-100,000 plants are planted per 667m², the electrophysiological indices, agronomic traits, yield and quality of *P. heterophylla* reached the best balance. The evaluation results of the comprehensive electrophysiological growth index and gray relational grade were highly consistent. Both of these studies confirmed that this planting density was beneficial, as shown in **Figure 9**. This consistency reflects the value of electrophysiological indicators as an early monitoring method. In plant growth, it can quickly reveal the physiological state of stressed plants, and predict the overall performance without harvesting. This further solves the problem of the lag of traditional agronomic trait evaluation, and provides a more efficient technology for the regulation of the precise cultivation density of *P. heterophylla*. These findings play a guiding role in the actual cultivation of *P. heterophylla*. Study the growth characteristics of *P. heterophylla* leaves and the laws of effective component accumulation, and provide a theoretical basis for the standardized cultivation of this medicinal plant.

**Figure 9:**
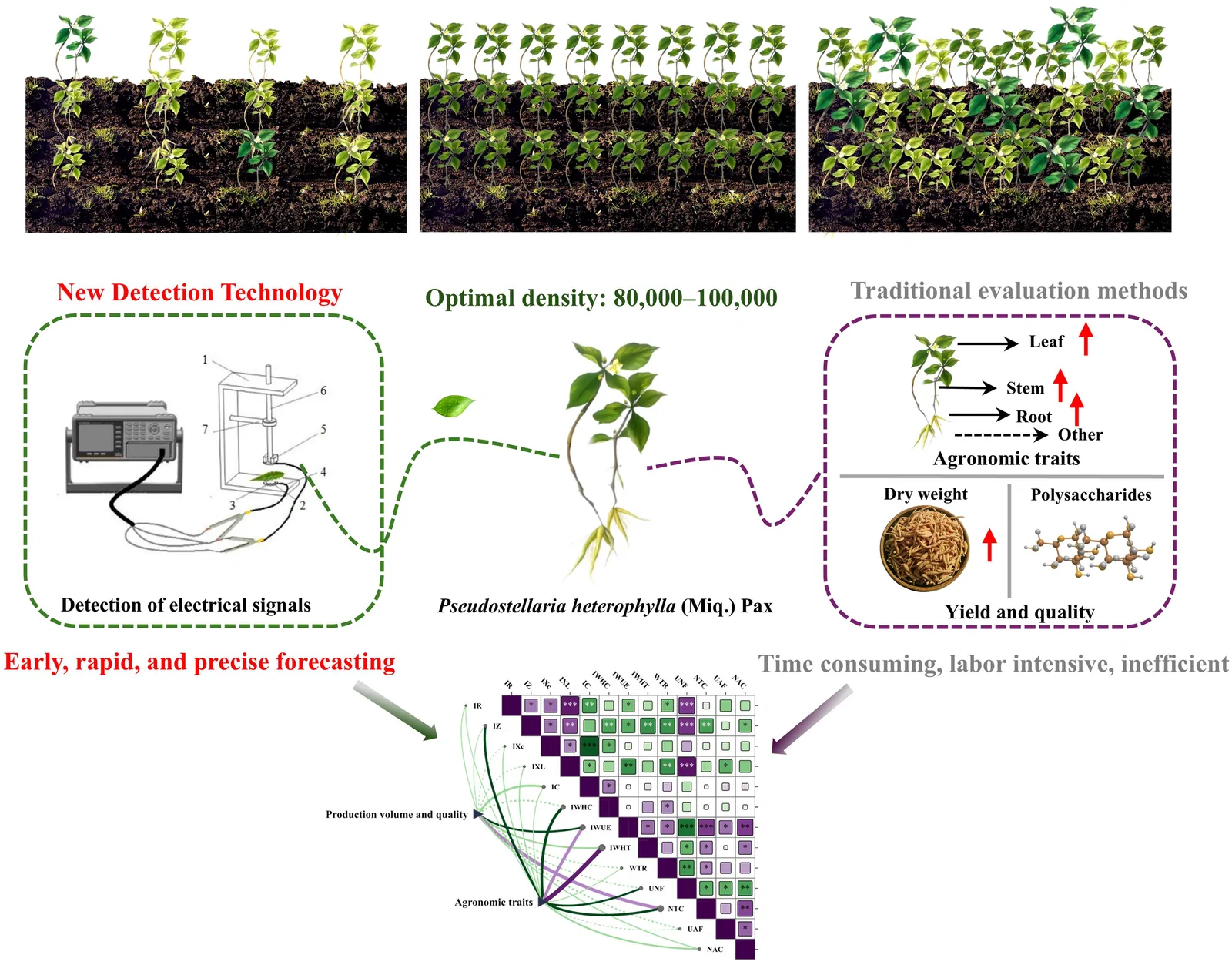
The response status of *P. heterophylla* to planting density in the context of electrophysiological and multi-trait association analysis.

It should be noted that, although the results of this study demonstrate a strong correlation between electrophysiological parameters and traditional growth indicators, the electrophysiology-derived indices (IWHC, IWHT, NTC, NAC, MA, etc.) are mathematically derived from base electrical parameters rather than directly measured physiological variables. While these indices are grounded in the bioenergetic framework established by Zhang et al. (2020, 2021c), no formal predictive model has yet been established, and the physiological interpretations of these indices remain to be validated by independent measurements (e.g., water potential, ion flux, metabolic profiling). Future work should focus on developing regression-based predictive models and conducting cross-validation using independent datasets, as well as incorporating direct physiological measurements to confirm the biological relevance of these electrophysiological indices.

Additionally, it should be emphasized that the conclusions of this study are based on a single cultivar, a single experimental site, a single growing season, and only three replicate plots per treatment. Given that plant responses to planting density can vary with cultivar, soil type, climatic conditions, and annual weather fluctuations, the generalizability of the recommended density range (80,000–100,000 plants per 667 m^2^) should be interpreted with caution. Multi-site, multi-cultivar, and multi-year validation studies are warranted to confirm the broader applicability of these findings.

## 5 Conclusion

This study innovatively combined electrophysiological signals with conventional evaluation methods to systematically elucidate the regulatory effects of different planting densities on the growth of *P. heterophylla*. Based on the comprehensive evaluation of grey correlation analysis, these signals show promise as potential early indicators of density stress in *P. heterophylla*. This study showed that a density of 80,000 to 100,000 plants per 667 m^2^ was the appropriate density for the growth of *P. heterophylla*. And the comprehensive performance of electrophysiological signals under this density was quite consistent with the traditional method. This study provides a scientific basis for the optimized cultivation of *P. heterophylla*, helps to improve yield or quality, promotes the development of a sustainable industry and economic growth. Further studies with independent validation datasets and formal model development are required to fully establish the predictive capability of these electrophysiological signals.

## Data Availability

The original contributions presented in the study are included in the article/supplementary material, further inquiries can be directed to the corresponding author/s.
